# Surgically cured paraneoplastic hypoglycemia associated with solitary fibrous tumor of the pleura: report of two cases

**DOI:** 10.1002/ccr3.1017

**Published:** 2017-05-31

**Authors:** Ryuta Fukai, Yoshihito Irie, Hiroyoshi Watanabe

**Affiliations:** ^1^ Department of General Thoracic SurgeryShonan Kamakura General HospitalKamakuraJapan; ^2^ Department of Cardiovascular SurgeryIwaki Kyoritsu General HospitalIwakiJapan; ^3^ Department of Respiratory MedicineDokkyo University Koshigaya HospitalKoshigayaJapan

**Keywords:** Paraneoplastic hypoglycemia, solitary fibrous tumor, surgery

## Abstract

Hypoglycemia is seldom seen in association with insulinomas, rare autoimmune diseases, and paraneoplastic situations. Paraneoplastic hypoglycemia is known as nonislet cell tumor‐induced hypoglycemia (NICTH). It is also known that a solitary fibrous tumor of the pleura can cause NICTH and that surgical resection is crucial to the success of NICTH treatment.

## Introduction

Hypoglycemia that is induced by insulin or oral hypoglycemic drugs is a common medical emergency in diabetic patients. However, hypoglycemia can be seen rarely in other conditions, such as insulinomas, rare autoimmune diseases, and paraneoplastic situations 1. Paraneoplastic hypoglycemia is known as nonislet cell tumor‐induced hypoglycemia (NICTH) 2; it was first reported as the Doege–Potter syndrome in 1930 3. It is also known that a solitary fibrous tumor of the pleura (SFTP) can cause NICTH. An SFTP is relatively rare; moreover, NICTH with SFTP happens in <5% of all pleural tumors 4. Therefore, the correct diagnosis of hypoglycemia associated with an SFTP may be difficult. We report two female NICTH cases with SFTP cured by surgery.

## Cases

### Case 1

**Figure 1:**
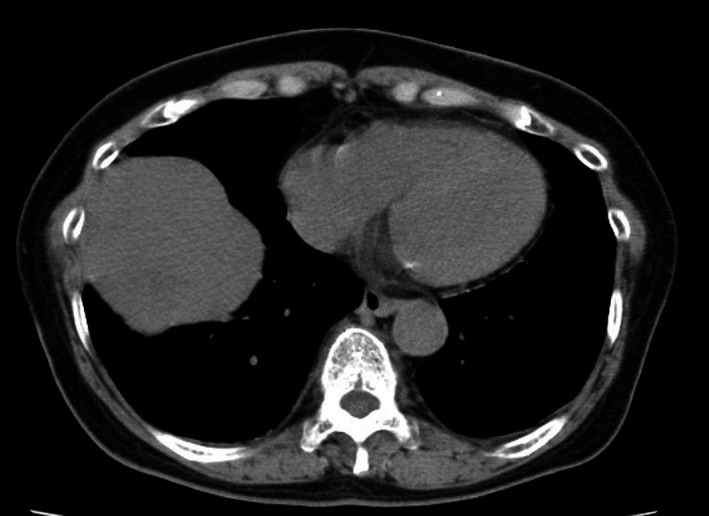
Computed tomography showed a homogenous, sharply defined mass, which was in contact with the middle lobe.

**Figure 2:**
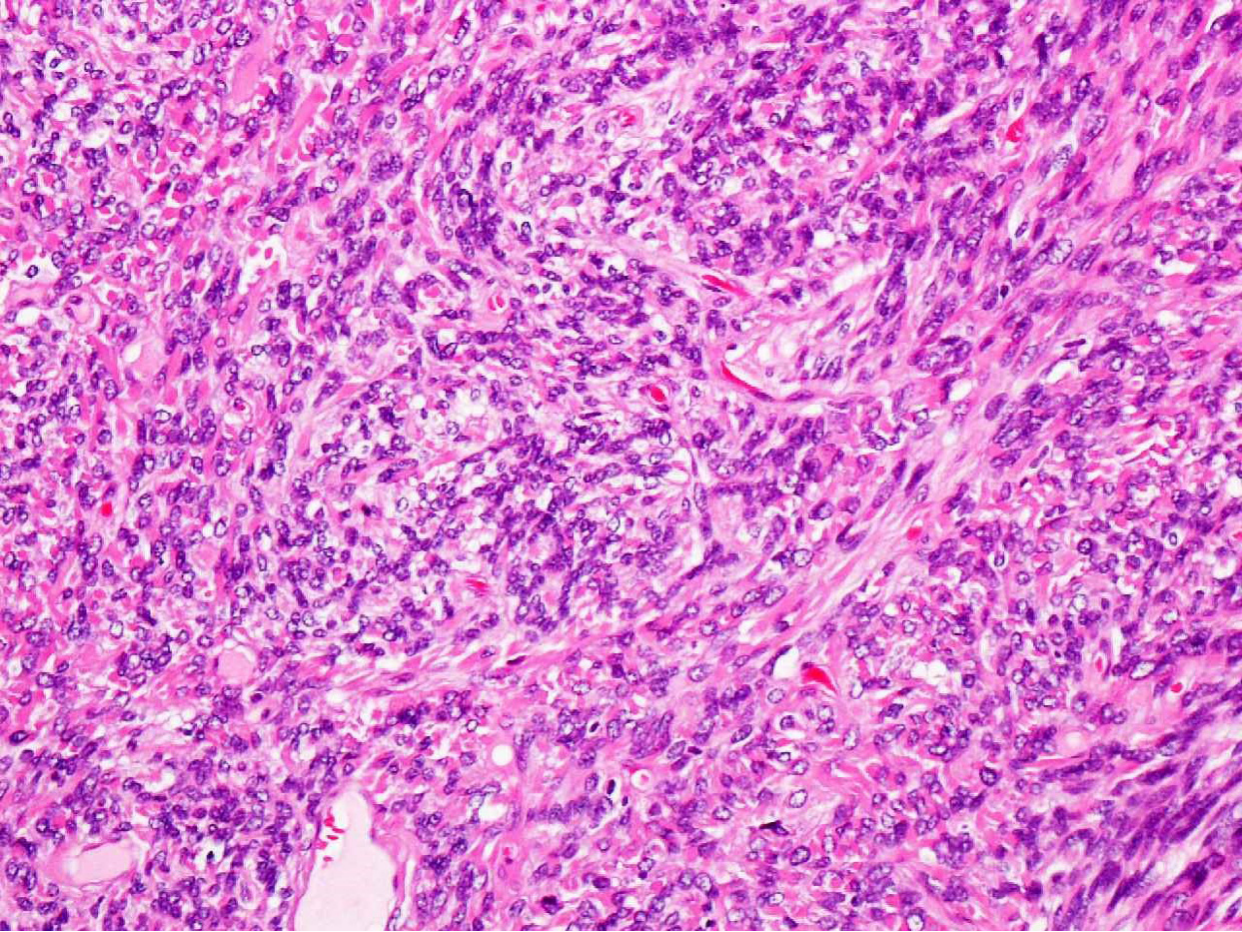
A microscopic specimen of the tumor showed bland, spindle cell proliferation that is patternless.

A 77‐year‐old woman was hospitalized due to dyspnea on exertion and repeated hypoglycemia with syncope. She had been diagnosed with a right solitary fibrous tumor through computed tomography (CT)‐guided biopsy 3 years previously. She had respiratory dysfunction (FEV_1.0_ 0.86L) because of bronchial asthma and a history of pulmonary tuberculosis. Because she had eaten at night to prevent hypoglycemic attacks, she had gained 8 kg over 6 months before entering our hospital. CT showed a well‐defined round tumor adjacent to the middle lobe (Fig. 1). We carefully carried out tumor extirpation with a right middle lobectomy through a posterolateral thoracotomy. The right middle lobe had almost collapsed under the pressure of the tumor. Bleeding was 1700 mL, and blood transfusion was needed. We also performed a tracheostomy after tumor resection for postoperative management. Her postoperative course was uneventful, and her hypoglycemic symptoms resolved just after the operation. Pathological examination revealed that the lesion was a solitary fibrous tumor (Fig. 2). The serum insulin‐like growth factor type II (IGF‐II) level, which was elevated preoperatively (869 ng/mL), returned to normal (269 ng/mL). She has been very well for 5 years after the operation (Table 1).

**Table 1:** Perioperative changes in laboratory findings and hormonal data associated with hypoglycemia

	Preoperative	Postoperative
Case 1		
Glucose (mg/dL)	51	91
HbA1c (%)	4.7	5.1
Insulin(*μ*U/mL)	<0.2	5.9
IGF‐2 (ng/mL)	869	269
Case 2		
Glucose (mg/dL)	42	106
HbA1c (%)	4.7	5.0
Insulin (*μ*U/mL)	0.1	12.5

### Case 2

**Figure 3:**
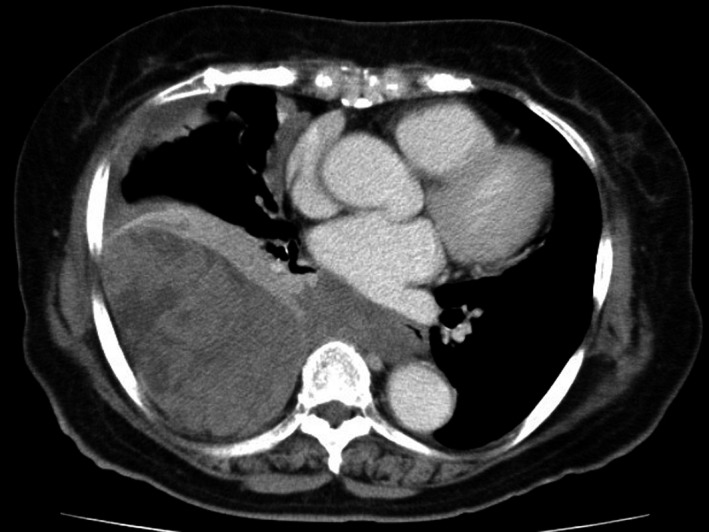
Computed tomography showed a large, well‐circumscribed mass with pleural effusions.

**Figure 4:**
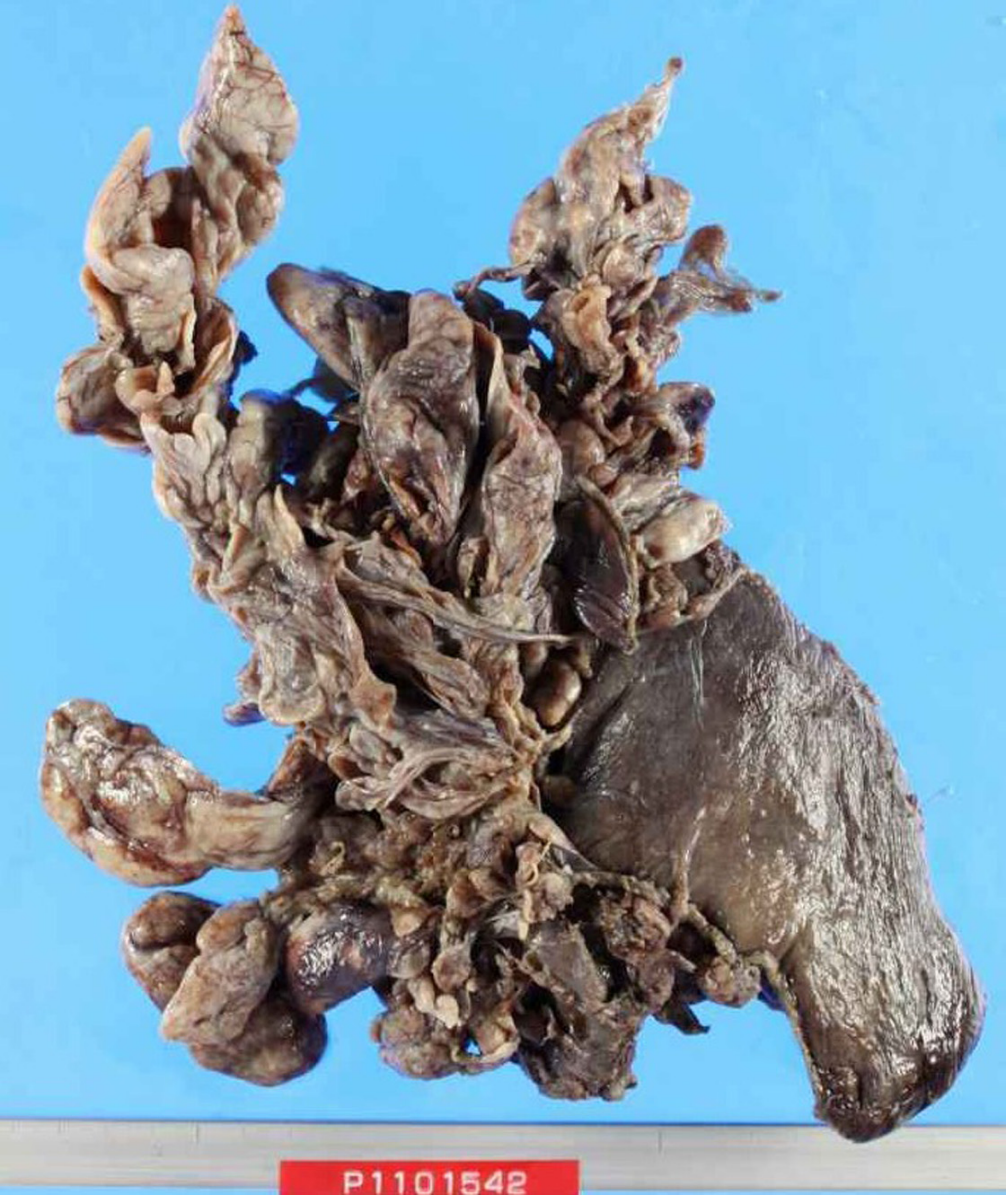
The resected tumor consisted of two components: a well‐defined mass in the right lower lobe and lesions that proliferated radially.

**Figure 5:**
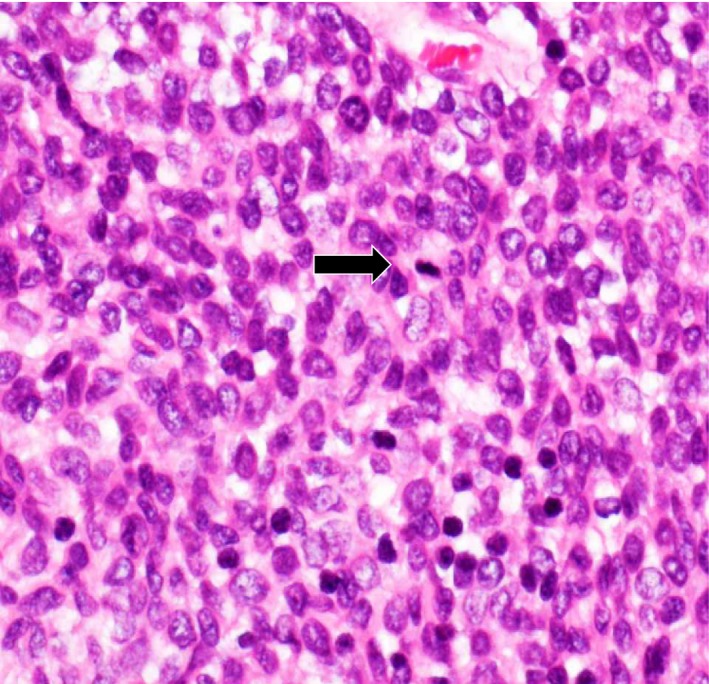
The tumor cells had high cellularity and prominent mitoses (arrow).

An 81‐year‐old woman visited our hospital because of cognitive symptoms during the period between getting out of bed and breakfast; moreover, her fasting blood glucose level was 41 mg/dL. She had a right pleural effusion on chest X‐ray, and CT showed a well‐demarcated huge mass, which pressed on the right lower lobe significantly (Fig. 3). Her serum insulin and growth hormone levels were low (0.1 *μ*U/mL and 0.11 ng/mL, respectively). We excised the right lower lobe with as much of the tumor as possible; the tumor had enlarged like the spokes of a wheel (Fig. 4). There were spindle cells with high cellularity and prominent mitoses in the specimen microscopically (Fig. 5). The cognitive disorder disappeared postoperatively. The patient has had no recurrence and remains in good condition 4 years after the operation.

## Discussion

Hypoglycemia, induced by antidiabetic treatment, is a common medical emergency in diabetic patients. However, hypoglycemia can be seen rarely in other conditions, such as insulinomas, rare autoimmune diseases, and paraneoplastic disorders 1. Paraneoplastic hypoglycemia is known as NICTH 2. NICTH with a pleural solitary fibrous tumor is rare, occurring in only approximately 5% of SFTP cases 4. Accordingly, it is difficult to diagnose correctly at the first consultation. Our first patient had had severe hypoglycemia with repeated syncope, although she had eaten excess foods at night. Therefore, she had received an intravenous glucose infusion day after day in another hospital. We had suspected the interrelation between hypoglycemia and SFTP from her symptoms, and then, we recognized NICTH. In the second patient with an SFTP, who had cognitive manifestations only in the early morning, the diagnosis of NICTH was easier because we had known it already. We performed a lobectomy and a tumor resection to the full extent possible. The patients have been well for several years after surgery with no recurrences.

Surgery is the first choice for effective treatment of NICTH, and completeness of the initial tumor resection is the key to preventing recurrence. Fortunately, our cases have had no recurrences; however, the symptoms may recur with recurrence of the lesion 5. It is reported that NICTH with hypoglycemia tends to be associated with remarkably large‐sized tumors and is associated with various malignant tumors, such as breast cancer, hepatic carcinoma, fibrosarcoma, as well as solitary fibrous tumors; therefore, we consider that they have a potentially malignant character. Actually, both a high cellularity and a high frequency of mitosis were observed in pathology specimens of the second case. These tumors manifesting as NICTH should be excised intact *en bloc* with clear margins, and intensive follow‐up is desirable. In our second case, the tumor had spread radially and extensively; therefore, we resected the tumor tissues as much as possible. Fortunately, this patient has been well with no recurrence for 4 years after the operation. If surgical resection is impossible, the effectiveness of glucocorticoid therapy has been reported 6.

NICTH is caused by secretion of a high‐molecular‐weight (HMW) form of IGF‐II 2. This HMW IFG‐II activates insulin receptors, thereby inhibiting hepatic gluconeogenesis and increasing peripheral glucose uptake, which results in hypoglycemia. Moreover, the HMW IGF‐II is able to bind to IGF‐I receptors leading to suppression of growth hormone by the pituitary, as well as reduction in insulin, IGF‐I, and IGF binding protein‐3 by the pancreas 3. Actually, in the first case, the increased preoperative serum IGF‐II level normalized postoperatively, and the serum insulin levels of both cases were low.

## Conclusion

We surgically treated two female NICTH patients with large solitary fibrous tumors. Their hypoglycemia improved immediately after surgery, and they have been well for four and five years after their operations with no recurrence. Surgical resection is the cornerstone of NICTH treatment.

## Authorship

All authors contributed to this work in this study. RF and YI: conducted these operations, and HW: performed clinical examinations. RF: wrote the manuscript chiefly.

## Conflict of Interest

None declared.
